# A bispecific antibody targeting conserved hemagglutinin epitopes confers broad protection against H7 avian influenza and accelerates viral clearance via localized respiratory delivery

**DOI:** 10.1080/22221751.2026.2713326

**Published:** 2026-08-19

**Authors:** Han Wu, Fan Yang, Ping Wang, Jiamin Fu, Jun Zhang, Jing Guo, Linfang Cheng, Fumin Liu, Nanping Wu, Hangping Yao, Haibo Wu

**Affiliations:** a State Key Laboratory for Diagnosis and Treatment of Infectious Diseases, National Clinical Research Center for Infectious Diseases, the First Affiliated Hospital, School of Medicine, Zhejiang University, Hangzhou, People’s Republic of China; b Department of Geriatrics, the Second Affiliated Hospital, School of Medicine, Zhejiang University, Hangzhou, People’s Republic of China; c Yuhang Institute for Collaborative Innovation and Translational Research in Life Sciences and Technology, Hangzhou, People’s Republic of China

**Keywords:** H7 avian influenza virus, hemagglutinin, bispecific antibody, broad protection, intranasal delivery

## Abstract

The continuous antigenic drift of zoonotic H7 avian influenza viruses remains a significant global public health threat. While monoclonal antibodies (mAbs) targeting the hemagglutinin (HA) head offer potent neutralization, their efficacy is often undermined by rapid viral escape. In this study, we systematically mapped the antigenic architecture of the H7 HA head domain using a panel of 30 murine mAbs. By integrating epitope mapping with longitudinal evolutionary analysis of over 2,500 isolates, we identified highly conserved, functionally constrained residues, specifically G70, G132, N167, and M173, that remained nearly invariant over decades. Leveraging these insights, we engineered a chimeric bispecific antibody (BsAb-H7) to simultaneously engage two distinct, non-overlapping conserved epitopes. BsAb-H7 demonstrated broad neutralization breadth across divergent H7N9, H7N7, and H7N3 subtypes *in vitro* and exhibited a superior *in vivo* pharmacokinetic profile. In lethal murine challenge models, BsAb-H7 provided robust prophylactic and therapeutic protection against heterologous H7N7 infection. Notably, localized intranasal administration significantly outperformed systemic delivery by accelerating viral clearance and resolving pulmonary immunopathology during delayed intervention windows. These results demonstrate that bispecific targeting of conserved HA epitopes effectively enhances antiviral breadth and limits immune escape, providing a promising strategy for the development of broadly protective therapeutics against rapidly evolving H7 influenza viruses.

## Introduction

Avian influenza virus (AIV), a negative-sense, single-stranded RNA virus of the Orthomyxoviridae family, possesses an eight-segmented genome that includes hemagglutinin (HA), neuraminidase (NA), and six additional gene segments [1]. Although most AIV subtypes cause limited human infection, the H7 subtype has demonstrated a notable capacity for zoonotic transmission, accounting for 1,687 confirmed human cases worldwide [2]. Since its first detection in humans in China in 2013, the H7N9 subtype has exhibited an unusual ability to cross species barriers [3,4]. Among more than 1,500 laboratory-confirmed human infections reported across five epidemic waves, 615 cases have resulted in death, corresponding to a case-fatality rate of 39% [5,6]. The persistent circulation and rapid evolution of H7 viruses continue to pose a significant threat to global public health [7].

Currently, vaccination remains the most effective strategy for influenza prevention [8], reducing disease risk by approximately 40–60% [9]. However, the high genetic variability of influenza viruses and the prolonged vaccine production timeline limit the ability to respond rapidly to emerging pandemic strains [10]. In contrast, antiviral agents targeting conserved viral components serve as essential first-line interventions by inhibiting viral replication, limiting transmission, and alleviating disease severity [11]. Standard treatment for H7 influenza primarily relies on NA inhibitors, such as oseltamivir and zanamivir [5,11]. However, their clinical efficacy is often suboptimal [12], as reflected by the persistently high case-fatality rate of over 30% observed in H7N9 infections [5]. This limited effectiveness is largely attributable to the emergence of antiviral resistance [13].

Therapeutic monoclonal antibodies (mAbs) targeting the HA glycoprotein have emerged as a promising modality for influenza treatment, owing to their high specificity and minimal off-target effects [14]. HA mediates viral entry through binding to sialic acid receptors followed by endocytosis [15], making it the principal target of neutralizing antibody responses. Structurally, HA consists of a globular head and a stem domain [16]. While stem-directed antibodies offer broad protection, they typically lack hemagglutination inhibition activity and rely more heavily on Fc-mediated effector functions for *in vivo* protection. Conversely, antibodies targeting the head domain mediate rapid and potent neutralization by directly blocking receptor binding [10]; however, their relatively constrained breadth restricts widespread utility. Bispecific antibody (BsAb), which simultaneously targets distinct, non-overlapping epitopes, offers a promising approach to enhance antiviral potency through synergistic neutralization while reducing the likelihood of viral escape [17]. This strategy has already been validated in multiple viral systems, including SARS-CoV-2 [18], HIV [19], Zika virus [20], dengue virus [21], and monkeypox virus [22].

In this study, we developed and evaluated a BsAb-based strategy to address the antigenic evolution of H7 influenza viruses. Rather than relying on single-epitope targeting, we employed a comprehensive panel of murine antibodies to identify highly conserved sites within the HA head domain and engineered a bispecific antibody (BsAb-H7) targeting two such epitopes. Our results demonstrate that BsAb-H7 provides robust prophylactic and therapeutic protection against lethal heterologous challenge. Furthermore, comparative analysis of administration routes revealed that localized intranasal (IN) delivery accelerates viral clearance more effectively than conventional systemic administration. These findings highlight a rational framework for the design of next-generation, broadly protective antibody-based therapeutics against rapidly evolving influenza viruses.

## Materials and methods

### Viruses, cell lines, and purified HA proteins

The H7 viruses used in this study included two H7N9 strains (A/Zhejiang/DTID-ZJU01/2013 and A/Guangdong/HP001/2017), one H7N7 strain (A/chicken/Jiangxi/C25/2014), and one H7N3 strain (A/duck/Zhejiang/DK10/2013). Additional influenza viruses comprised A/Michigan/45/2015 (H1N1), A/duck/Zhejiang/6D10/2013 (H2N8), A/Hong Kong/2671/2019 (H3N2), A/Texas/37/2024 (H5N1), A/chicken/Zhejiang/1664/2017 (H6N1), A/duck/Zhejiang/D4/2018 (H9N2), A/Zhejiang/CNIC-ZJU01 /2023 (H10N5), and B/Phuket/3073/2013 (influenza B virus). All viral strains were preserved in our laboratory as previously described [23,24] (Table S1). Viruses were propagated in 9-day-old specific pathogen-free (SPF) embryonated chicken eggs and titrated using the 50% tissue culture infectious dose (TCID_50_) method [25].

H7 AIV antigens (H7-Re 3 and H7-Re 4) were purchased from Harbin Guosheng Biotechnology (Harbin, China). Madin–Darby canine kidney (MDCK) cells and SP2/0 mouse myeloma cells were maintained under standard culture conditions in our laboratory. Purified HA proteins derived from multiple H7 strains, including A/Zhejiang/DTID-ZJU01/2013, A/duck/Zhejiang/DK10/2013, A/chicken/Jiangxi/C25/2014, A/Guangdong/HP001/2017, A/chicken/Inner Mongolia/SD010/2019, A/chicken/Yunnan/SD024/2021, and A/chicken/Hebei/QG2/2023, were obtained from Sino Biological Inc. (Beijing, China). In addition, a split-virion H7 vaccine (30 μg/mL HA, A/ZJU01/PR8/2013) produced by Zhejiang Tianyuan Bio-Pharmaceutical Co., Ltd. (Hangzhou, China) was used as the immunogen for mouse immunization protocols [26].

### Generation and isotyping of H7 mAbs

**Figure 1.**
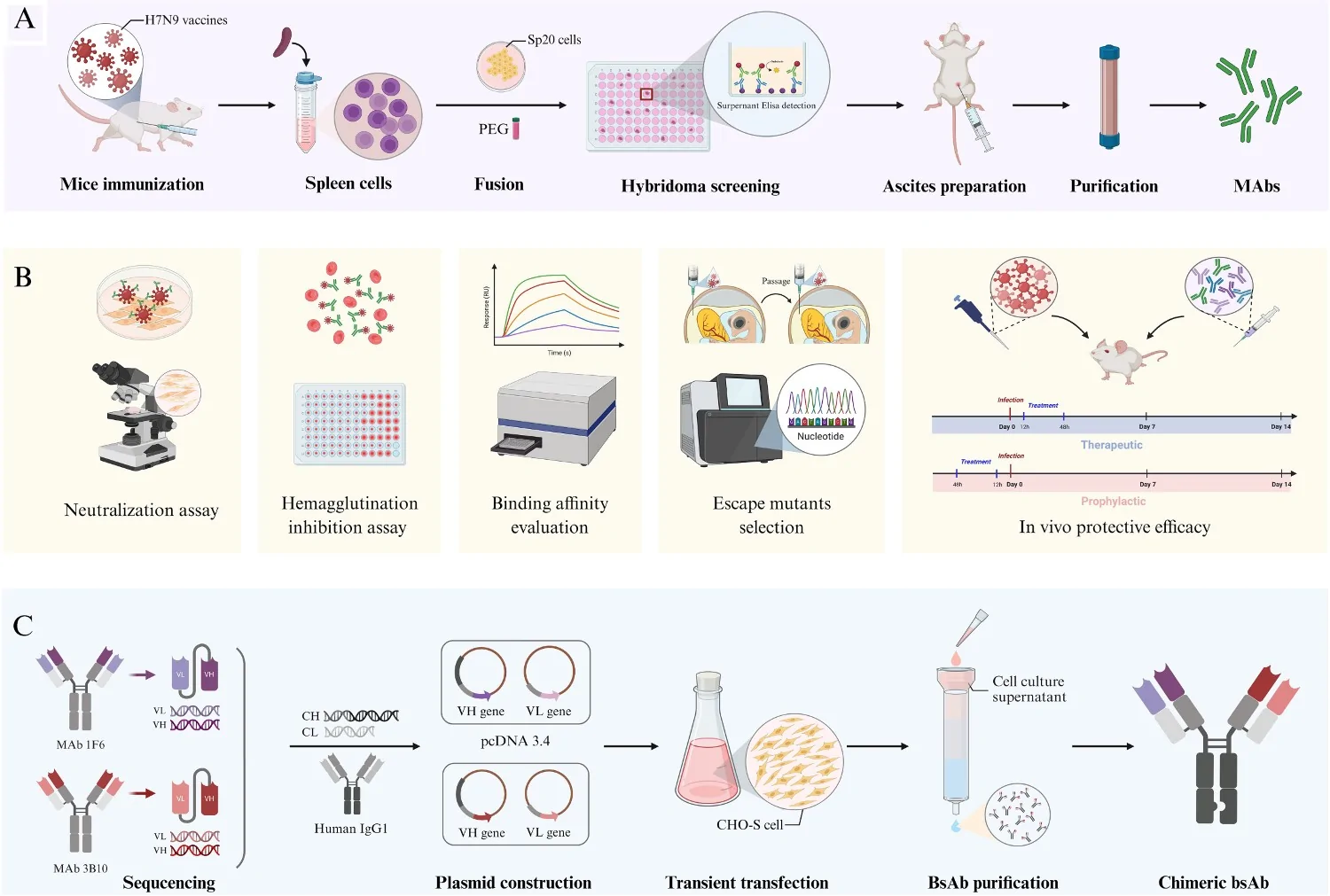
Integrated pipeline for generating and characterizing H7-targeting monoclonal and bispecific antibodies. (A) Schematic overview of murine mAb generation against H7 influenza viruses, in which mice were immunized with H7N9 vaccine to induce antigen-specific B cells, followed by isolation of splenocytes and fusion with SP2/0 myeloma cells using polyethylene glycol; hybridomas were subsequently screened by ELISA for H7N9 reactivity, and positive clones were expanded for *in vivo* ascites production and antibody purification. (B) Functional characterization workflow of selected mAbs, including neutralization assays to assess viral inhibition in cell culture, hemagglutination inhibition assays to evaluate blockade of virus-induced red blood cell agglutination, and binding affinity measurements by surface plasmon resonance or ELISA; escape mutants were generated through serial viral passage under mAb selection pressure, and protective efficacy was evaluated in mouse models under both prophylactic and therapeutic regimens. (C) Construction of the chimeric BsAb-H7, in which the variable regions (VH and VL) of two murine mAbs (1F6 and 3B10) were sequenced and cloned into pcDNA3.4 vectors containing human IgG1 constant domains (CH and CL), followed by transient transfection into CHO-S cells for recombinant expression; the BsAb-H7 was subsequently purified from culture supernatants and characterized as a dual-specific antibody targeting distinct H7 epitopes.

Five six-week-old female BALB/c mice were obtained from SLAC Laboratory Animal Co., Ltd. (Shanghai, China) to establish a hybridoma-based mAb generation pipeline (Figure 1A), as previously described [24]. Mice were immunized via intramuscular injection of H7N9 vaccine (A/ZJU01/PR8/2013, 200 μL per dose) following a prime–boost regimen at two-week intervals. A final booster immunization was administered three days prior to cell fusion to enhance B-cell activation. Splenocytes were subsequently harvested and fused with SP2/0 mouse myeloma cells using polyethylene glycol (PEG)-mediated fusion at 37 °C. Hybridomas were cultured in hypoxanthine–aminopterin–thymidine (HAT) selection medium, and culture supernatants were screened by antigen-specific enzyme-linked immunosorbent assay (ELISA). Positive clones were subjected to three rounds of limiting dilution to ensure monoclonality.

Monoclonal antibodies were purified from murine ascites using Protein G affinity chromatography (GE Healthcare, Chicago, IL, USA), aliquoted, and stored at −80 °C. Antibody isotypes were determined using a mouse mAb isotyping kit (Bio-Rad Laboratories, Hercules, CA, USA). Variable region sequences of the heavy and light chains were obtained from Sino Biological Inc. As illustrated in Figure 1B, a systematic characterization workflow was applied to select candidate antibodies with high affinity, favourable pairing compatibility, and strong epitope conservation for subsequent BsAb engineering.

### ELISA evaluation

ELISA was performed as previously described [24]. Briefly, 96-well microplates were coated overnight at 4 °C with purified H7 HA protein (20 ng per well). After blocking with 200 μL of blocking buffer for 2 h at room temperature, mAbs were initially diluted to 10 μg/mL and subjected to two-fold serial dilutions in phosphate-buffered saline (PBS). A volume of 100 μL of each dilution was added to the wells and incubated to allow antigen–antibody binding. Following washing steps, bound antibodies were detected using horseradish peroxidase (HRP)-conjugated goat anti-mouse IgG secondary antibody (Novus Biologicals, Centennial, CO, USA). After substrate development, absorbance was measured at 450 nm using a microplate reader (Bio-Rad Laboratories) to assess binding activity. In addition, hybridoma culture supernatants (100 μL per well) were screened using the same ELISA protocol.

### *In vitro* hemagglutination inhibition (HI) and microneutralization (MN) assays

HI and MN assays were performed as previously described [23]. For the HI assay, viral strains A/Zhejiang/DTID-ZJU01/2013 (H7N9), A/chicken/Jiangxi/C25/2014 (H7N7), and A/duck/Zhejiang/DK10/2013 (H7N3) were standardized to 4 hemagglutination units (HAU). These standardized viruses were pre-incubated with two-fold serial dilutions of mAbs in V-bottom-96-well plates for 20 min at room temperature. Subsequently, 1% (v/v) chicken red blood cells were added, and the HI titre was defined as the highest antibody dilution that completely inhibited hemagglutination. To ensure the reliability and reproducibility of the data, all HI assays were evaluated in duplicate wells per run and were completely repeated across three independent experiments.

For the MN assay, mAbs were initially diluted to 100 μg/mL and subjected to two-fold serial dilutions, followed by incubation with 100 TCID_50_ of H7 virus for 2 h. The antibody–virus mixtures were then added in duplicate to confluent MDCK cell monolayers. Cytopathic effects (CPE) were monitored microscopically, and the half-maximal inhibitory concentration (IC_50_) values were calculated using the Reed–Muench method to ensure inoculum consistency [27]. Dose–response curves were fitted using nonlinear regression. All data analyses were performed using GraphPad Prism 10 software (GraphPad Software, San Diego, CA, USA).

### Immunofluorescence assay (IFA)

IFA was conducted to evaluate the specificity of mAbs as previously described [24]. MDCK cells were seeded in 96-well plates at a density of 4 × 10^4^ cells per well and infected with virus at a multiplicity of infection (MOI) of 0.5. After 24 h of incubation, cells were fixed with 4% paraformaldehyde and permeabilized with 0.5% Triton X-100 for 30 min at 25 °C. Following blocking, mAbs were added at a concentration of 10 μg/mL and incubated accordingly. Bound antibodies were detected using Alexa Fluor 488-conjugated goat anti-mouse IgG (Abcam, Cambridge, MA, USA) after 1 h incubation at 37 °C. Nuclei were counterstained with 4’,6-diamidino-2-phenylindole (DAPI). Fluorescence images were acquired using a confocal laser scanning microscope (Leica TCS SP8; Leica Microsystems, Wetzlar, Germany).

### RNA isolation and reverse transcription quantitative real-time polymerase chain reaction (RT-qPCR)

RNA extraction and RT-qPCR were performed as previously described [28]. Briefly, total cellular RNA was isolated using Nucleic Acid Extraction Reagent on an automated EX3600 Nucleic Acid Extraction System (Liferiver Bio-Tech Corp., Shanghai, China). Complementary DNA (cDNA) was synthesized from the purified RNA and subsequently subjected to RT-qPCR using the Influenza A Virus Nucleic Acid Detection Kit (Liferiver Bio-Tech Corp.), according to the manufacturer’s instructions. Amplification and real-time fluorescence detection were carried out using a CFX96 Real-Time PCR Detection System (Bio-Rad Laboratories, Hercules, CA, USA).

### Isolation and characterization of escape mutations

Escape mutants were generated by serial passaging of H7N9 viruses under increasing concentrations of mAbs, as previously described [29]. Briefly, each mAb was diluted to 2 and 4 ng/mL and pre-incubated with 100 TCID_50_ of H7N9 virus (A/Zhejiang/DTID-ZJU01/2013) for 1 h at 37 °C. The mixture was then inoculated into 9-day-old SPF embryonated chicken eggs and incubated at 35 °C for 72 h. Allantoic fluids were harvested and screened by hemagglutination assay. Positive fluids were subjected to a second passage under identical conditions, with mAb concentrations escalated to 8 and 16 ng/mL to maintain selective pressure. Viral RNA was extracted from positive fluids and the HA gene was amplified and sequenced. Mutation frequency was monitored by comparing Sanger sequencing chromatograms across passages, and consensus amino acid substitutions were confirmed when variant signals exceeded 50% of the total signal. Finally, the identified mutations were mapped onto the H7 HA crystal structure (retrieved from the Protein Data Bank) using PyMOL (v2.5.0; Schrödinger, New York, NY, USA) [30] to define potential antibody-binding epitopes. Throughout this manuscript, HA residues are numbered according to the H3 subtype convention (H3 numbering), with the signal peptide explicitly retained in the positional scheme.

### Model prediction and docking

The fragment variable (Fv) domain structures of the BsAb-H7 were computationally predicted using ABodyBuilder2 [31]. The predicted models were subsequently subjected to protein–protein docking using ZDOCK [32] and ClusPro 2.0 [16]. The top five docking complexes, ranked by ZRANK [33] and ClusPro multi-mode clustering (balanced, electrostatic, hydrophobic, and van der Waals/electrostatic), were systematically screened. Representative models exhibiting the highest structural consistency were selected for visualization and structural analysis using UCSF ChimeraX (v1.6; University of California, San Francisco, CA, USA) [34].

### *In vivo* pharmacokinetic evaluation

Pharmacokinetic profiles of the BsAb-H7 and parental mAbs were evaluated in six-week-old male BALB/c mice (n = 5) according to previous study [35]. Mice received a single intraperitoneal (IP) injection of antibodies at a dose of 10 mg/kg. Serum samples were collected longitudinally over a 40-day period (days 1-40), and antibody concentrations were determined by ELISA using H7 HA-coated plates, with detection performed using an HRP-conjugated secondary antibody. Pharmacokinetic parameters were calculated using GraphPad Prism 10 software and non-compartmental analysis (NCA) using Phoenix WinNonlin 8.3 (Certara, USA). The statistical differences were assessed using one-way ANOVA followed by Tukey's *post-hoc* test.

### Phylogeography and sequence analysis

To evaluate the evolutionary conservation and variability of escape mutation sites, a global dataset comprising 5,504 full-length H7 HA amino acid sequences from influenza viruses, collected from diverse avian and mammalian hosts up to March 2026, was retrieved from the GISAID database. Multiple sequence alignment was performed using MAFFT (v7.0) [36], and residue variation at epitope sites was analyzed and visualized using Jalview [37]. Alignment positions with ≥95% amino acid identity were defined as highly conserved. This threshold is more stringent than the ≥85% default applied in Gblocks and aligns with established criteria for identifying functionally conserved residues [38,39]. Phylogenetic analysis was conducted using representative HA gene sequences sampled between 1988 and 2025, employing the neighbor-joining method implemented in MEGA (v11) [40]. Branch support was assessed with 1,000 bootstrap replicates. Viral clades were defined using a 95% nucleotide sequence identity threshold to ensure consistent lineage classification within the H7 phylogeny. To identify sites potentially under immune-driven selection, epitope residues exhibiting >5% frequency variation across surveillance periods were selected for statistical testing. Monotonic changes in temporal frequency were evaluated via the Cochran–Armitage trend test, whereas non-monotonic fluctuations were assessed using Pearson's χ² test. Two-sided *P*-values <0.05 were considered statistically significant. All computations were implemented in R (v4.3.0, R Foundation for Statistical Computing, Vienna, Austria).

### Construction and generation of chimeric BsAb-H7

As illustrated in Figure 1C, the variable regions (VH and VL) of the BsAb-H7 were derived from parental mAbs 1F6 and 3B10. Correct chain pairing was achieved using the CrossMab CH1–CL platform [41], while heterodimerization was facilitated by the knobs-into-holes strategy [14]. The variable domain sequences were obtained from Sino Biological Inc., synthesized, and cloned into a human IgG1 backbone to generate chimeric constructs (GenScript Biotech Corp., Nanjing, China) [42].

The constructs were expressed in suspension-adapted CHO-S cells cultured in serum-free medium at 36.5 ± 0.5 °C with 5% CO_2_. Culture supernatants were harvested when cell viability dropped below 80%, clarified, and subjected to protein affinity chromatography for purification. The purified antibodies were analyzed for purity, integrity, and homogeneity using SDS-PAGE, and quantified by measuring absorbance at 280 nm.

### *In vivo* prophylactic and therapeutic protection

To establish the lethal challenge model, the 50% mouse lethal dose (MLD_50_) was determined by inoculating six-week-old male BALB/c mice (SLAC Laboratory Animal Co., Ltd., Shanghai, China) with serial dilutions of virus in a 60 μL volume, as previously described [24]. The *in vivo* protective efficacy of antibodies was subsequently evaluated under both prophylactic and therapeutic regimens using the A/chicken/Jiangxi/C25/2014 (H7N7) virus [43]. Survival and body weight were monitored daily for 14 days. In accordance with animal ethics guidelines, mice exhibiting ≥25% body weight loss were humanely euthanized.

For prophylactic studies, mice (n = 11 per group) received mAbs at doses of 1 or 10 mg/kg in a total volume of 200 μL via IP injection at 12 and 48 h prior to viral challenge. Mice were then challenged intranasally with 5 × MLD_50_ of virus in 80 μL. For therapeutic studies, mice (n = 11 per group) were first infected intranasally with 5 × MLD_50_ of virus (80 μL), followed by administration of mAbs at doses of 1, 3, or 10 mg/kg via IP injection at 12 and 48 h post-infection (hpi). Isotype control antibodies (10 mg/kg; Solarbio Life Sciences, Beijing, China) were included in both experimental settings. Sample sizes were determined based on previous studies [24].

To assess viral pathogenesis, n = 3 mice from each group were euthanized at 3 and 6 days post-infection (dpi). Organs, including the heart, lungs, spleen, kidneys, and liver, were collected for viral titration and load quantification. Viral titres in lung homogenates were determined by TCID_50_ assay and log_10_-transformed prior to analysis were compared across groups using one-way ANOVA with Tukey's HSD *post-hoc* test. Longitudinal body weight changes and cumulative viral load kinetics were evaluated via a two-way ANOVA followed by Tukey’s multiple comparisons test. Survival outcomes were analyzed using the Kaplan-Meier method and the log-rank (Mantel–Cox) test. Concurrently, a subset of lung tissue was reserved and fixed for histopathological analysis [24]. Statistical evaluations were performed using GraphPad Prism 10, and significance was defined by two-sided *P*-values < 0.05.

### Intranasal administration of BsAb-H7

Following intranasal infection with 5 × MLD_50_ of H7N7 virus in 80 μL PBS, mice (n = 11 per group) were treated with BsAb-H7 via the intranasal route at doses of 1 or 10 mg/kg. Treatments were administered at 12 h or 48 hpi, consistent with the intraperitoneal dosing schedule. Body weight was recorded daily to monitor disease progression. At 3 and 6 dpi, subsets of mice (n = 3 per time point) were euthanized, and lung tissues were harvested to assess viral load and histopathological changes. Sample processing and analysis were performed using the same procedures as described for the intraperitoneal treatment groups.

### Surface plasmon resonance (SPR)

The binding affinity of BsAb-H7 to recombinant H7 HA was measured by SPR using a Biacore 8 K system (GE Healthcare). Briefly, BsAb-H7 was immobilized onto a CM5 sensor chip in HBS-EP running buffer. Recombinant H7 HA antigens were injected in triplicate using a two-fold serial dilution series spanning 0.39–100 nM. The association and dissociation phases were set to 180 and 300 s, respectively, followed by chip regeneration with 10 mM glycine-HCl (pH 1.5). Binding kinetics were analyzed to determine the association rate (k_a_), dissociation rate (k_d_), and equilibrium dissociation constant (K_D_). Data were processed using Biacore Evaluation Software (v3.0; GE Healthcare) and fitted to a 1:1 Langmuir binding model as previously described [44].

## Results

### Characterization of generated mabs

#### Binding affinities against purified H7 HA proteins

**Figure 2.**
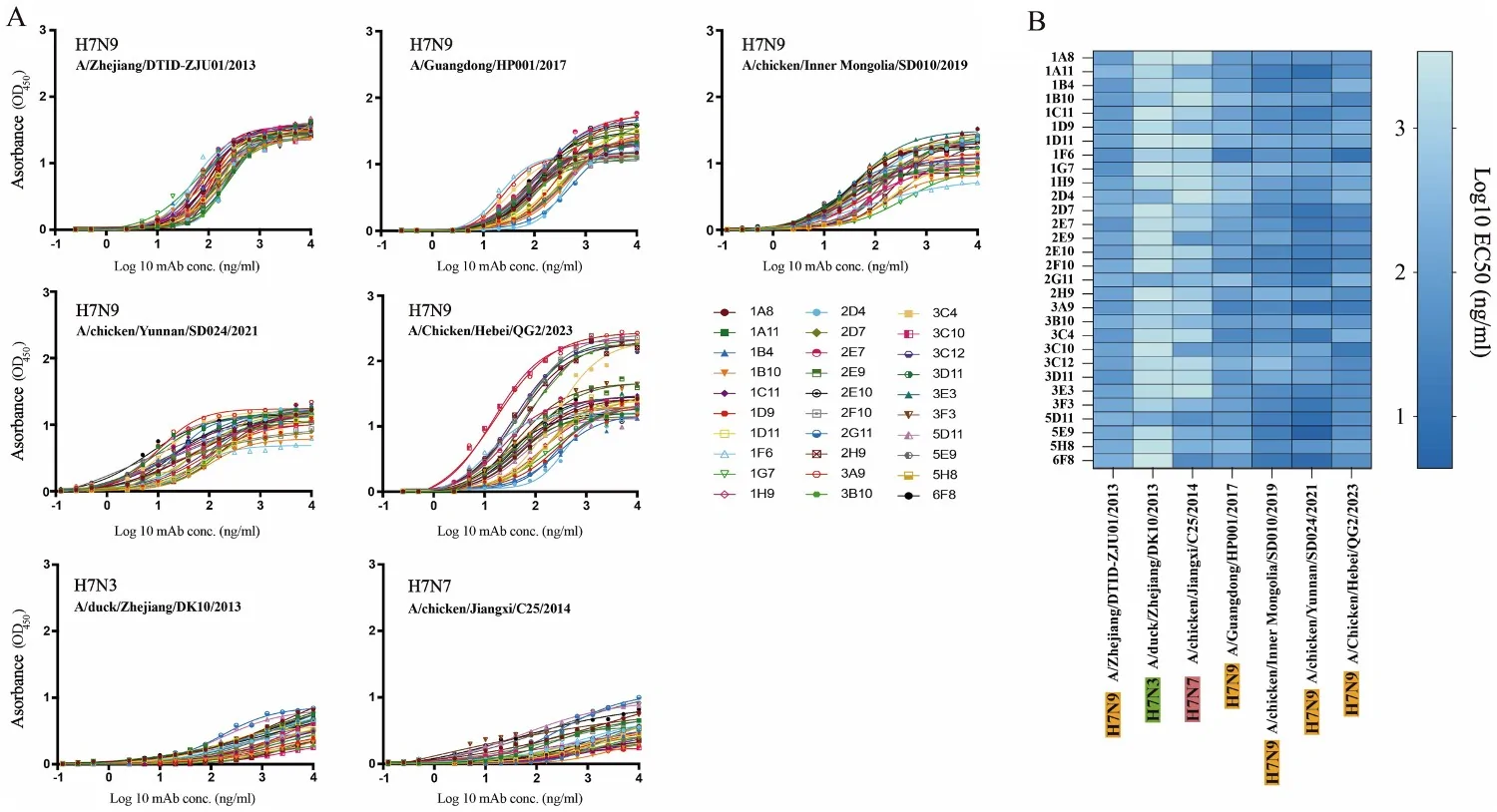
Binding profiles of 30 mAbs against HA proteins of diverse H7 influenza viruses. (A) Dose–response binding curves of 30 mAbs against HA proteins derived from multiple H7 strains, including H7N9 (A/Zhejiang/DTID-ZJU01/2013, A/Guangdong/HP001/2017, A/chicken/Inner Mongolia/SD010/2019, A/chicken/Yunnan/SD024/2021, and A/chicken/Hebei/QG2/2023), H7N3 (A/duck/Zhejiang/DK10/2013), and H7N7 (A/chicken/Jiangxi/C25/2014). The diverse colours and symbols in the central legend distinguish the 30 individual mAbs. (B) Heatmap showing the log_10_-transformed EC_50_ values (ng/mL) of each mAb against the corresponding HA proteins. The colour scale on the right indicates binding affinity, where darker blue represents lower EC_50_ values. Along the horizontal axis, the coloured background boxes denote specific viral subtypes: H7N9 in orange, H7N3 in green, and H7N7 in maroon.

A panel of 30 hybridomas producing murine mAbs against H7 HA was successfully generated. As summarized in Table 1, the monoclonal antibody panel comprised predominantly IgG isotypes, with two exceptions of the IgM class. The IgG antibodies were distributed across three subclasses (IgG2a, IgG2b, and IgG1), and all clones uniformly employed the κ light chain. To assess binding profiles, ELISA was performed using purified HA proteins from seven H7 strains representing multiple subtypes (H7N9, H7N3, and H7N7), avian hosts, and isolation years (2013–2023). As shown in Figure 2, all 30 mAbs exhibited broad binding activity across the tested HA proteins. This binding to H7N9 HA remained highly consistent over time, with most mAbs retaining strong affinity (half-maximal effective concentration (EC_50_) < 0.5 μg/mL) for isolates spanning 2013–2023, indicating robust recognition of both historical and recent strains. In contrast, certain mAbs, such as 1D9 and 1G7, showed preferential binding to early H7N9 strains but markedly reduced affinity for more recent variants. Although binding to H7N3 and H7N7 HAs was generally weaker than that to H7N9, six mAbs (1F6, 2G11, 3A9, 3B10, 3F3, and 5D11) maintained effective binding to both subtypes, with EC_50_ values below 1 μg/mL.

**Table 1.** Characterization of 30 mAbs.

No.	Clone	Isotype	HI activities (μg/mL)			MN activities (μg/mL)		
			H7N9	H7N7	H7N3	H7N9	H7N7	H7N3
1	1A8	IgG2a, κ	0.4	15.6	>1000	0.78	3.125	0.78
2	1C11		1000	>1000	>1000	1.56	100	100
3	2D7		1000	>1000	>1000	0.19	0.39	0.39
4	2E10		50	>1000	>1000	6.25	>100	>100
5	3C4		250	>1000	>1000	0.78	>100	>100
6	3E3		**8**	**15.6**	**15.6**	**0**.**19**	**0**.**39**	**0**.**39**
7	3F3		**0.8**	**15.6**	**15.6**	**0**.**39**	**0**.**39**	**0**.**39**
8	5E9		3	125	>1000	0.39	0.78	0.39
9	1A11	IgG2b, κ	1000	>1000	>1000	25	100	3.125
10	1B4		4	15.6	>1000	0.19	0.39	0.39
11	1F6		**3**	**125**	**250**	**25**	**6**.**25**	**1**.**56**
12	2E7		1000	>1000	>1000	1.56	>100	>100
13	2E9		**6**	**125**	**250**	**3**.**125**	**12**.**5**	**0**.**39**
14	2F10		3	125	>1000	0.78	50	3.125
15	3A9		250	15.6	>1000	100	0.39	0.39
16	5D11		4	15.6	>1000	0.39	0.39	0.39
17	6F8		0.1	15.6	>1000	0.39	6.25	100
18	1B10	IgG1, κ	500	>1000	>1000	3.125	>100	>100
19	1D11		1000	>1000	>1000	50	>100	>100
20	1D9		4	15.6	>1000	0.19	0.39	0.39
21	1G7		1000	>1000	>1000	50	100	100
22	1H9		62.5	500	>1000	0.39	100	12.5
23	2H9		**250**	**15.6**	**15.6**	**12**.**5**	**0**.**78**	**0**.**39**
24	3B10		**125**	**250**	**15.6**	**50**	**1**.**56**	**0**.**39**
25	3C10		**62.5**	**62.5**	**500**	**100**	**0**.**39**	**0**.**39**
26	3C12		0.8	15.6	>1000	0.78	1.56	1.56
27	3D11		250	1000	>1000	12.5	50	0.39
28	5H8		**8**	**125**	**250**	**1**.**56**	**1**.**56**	**0**.**78**
29	2D4	IgM	25	1000	>1000	1.56	100	100
30	2G11		62.5	500	>1000	6.25	50	12.5

Note: Mouse mAb isotypes and subclasses were determined using a commercial isotyping kit. HI and MN assays were performed using the following H7 strains: A/Zhejiang/DTID-ZJU01/2013 (H7N9), A/chicken/Jiangxi/C25/2014 (H7N7), and A/duck/Zhejiang/DK10/2013 (H7N3). Antibodies with strong functional activity are defined as those exhibiting HI titres < 1000 μg/mL and neutralizing potency (IC_50_) < 100 μg/mL, which was highlighted in bold with a grey background.

#### HI activities against H7 viruses

As shown in Table 1, all 30 mAbs exhibited HI activity against H7N9 (A/Zhejiang/DTID-ZJU01/2013), with titres ranging from 0.1 to 10^3^ μg/mL. When evaluated against H7N7 (A/chicken/Jiangxi/C25/2014), most mAbs demonstrated potent inhibition, with HI titres ≤15.6 μg/mL. Only six mAbs (1B10, 3C4, 2E10, 2E7, 1A11, and 1D11) showed no detectable HI activity against this strain. In contrast, activity against H7N3 (A/duck/Zhejiang/DK10/2013) was markedly limited, with only eight mAbs retaining measurable HI activity. Notably, these mAbs (2H9, 3B10, 3C10, 3E3, 3F3, 2E9, 1F6, and 5H8) demonstrated cross-subtype HI activity against H7N9, H7N7, and H7N3, suggesting their ability to target functionally conserved epitopes within the HA head domain.

#### MN activities against H7 viruses

The neutralizing activity of the mAbs was systematically evaluated against multiple H7 subtypes (Table 1). All mAbs effectively neutralized H7N9, with the most potent achieving inhibition at concentrations as low as 0.19 μg/mL, and approximately half exhibiting strong activity below 3.125 μg/mL. In contrast, mAbs 1B10, 3C4, 2E10, 2E7, and 1D11 failed to neutralize either H7N7 or H7N3 within the tested concentration range. Importantly, a subset of 25 mAbs, including 1A8, 1A11, 1B4, 1D9, 1F6, 1H9, 2E9, 2F10, 2G11, 2H9, 3A9, 3B10, 3C10, 3C12, 3D11, 3E3, 3F3, 5D11, 5E9, 5H8, and 6F8, demonstrated broad cross-neutralizing activity against H7N9, H7N7, and H7N3, with effective concentrations ranging from 0.19 to 100 μg/mL. Integration of HI and MN data further identified eight mAbs (2H9, 3B10, 3C10, 3E3, 3F3, 2E9, 1F6, and 5H8) as consistently potent and broadly reactive, highlighting their potential as candidates for BsAb development.

### Conservation of mAb epitopes in H7 HA

In this study, twelve distinct escape mutation sites were identified through selective pressure experiments. Among these, nine mutants harboured single amino acid substitutions in HA, while three contained double substitutions. To evaluate the evolutionary constraints at these sites, a global dataset comprising 2,667 non-redundant H7 HA sequences retrieved from GISAID (up to March 2026) was aligned using MAFFT for comprehensive conservation analysis. The frequency of amino acid variants at each position was used to assess their prevalence among circulating avian influenza viruses.

Analysis of escape mutants selected by 30 mAbs revealed 12 distinct epitopes, with seven mAbs associated with double-site mutations (Table 2). A recurrent mutational pattern, G151E and R148M, conferred resistance to five mAbs (1A8, 1B4, 2F10, 3F3, and 5E9). In contrast, the 1D11 escape mutant exhibited a combination of G151E and E113 K substitutions, whereas the 2E10 mutant harboured G214E and R65 K substitutions. Four residues (G70, G132, N167, and M173) were identified as key epitopes targeted by mAbs 1A11, 1H9, a subset including 3B10 (together with 2H9, 3D11, and 3A9), and 1F6, respectively. Notably, these residues displayed remarkable evolutionary conservation, with frequencies consistently exceeding 95% across all time periods analyzed, suggesting strong functional or structural constraints. Among them, N167 was particularly conserved, with the N167D substitution occurring at a very low frequency (0.3-0.6%), indicating strong purifying selection.

**Table 2.** Assessments of amino acid substitutions at HA positions targeted by mAbs.

No.	Location	mAb(s)	Prevalence of amino acid mutations in multiple H7 strains (%)			
			Pre-2012	2013–2016	2017–2025	Total
			(n = 784)	(n = 890)	(n = 993)	(n = 2667)
1	G70S	1A11	G (96%, 753)	G (97.9%, 871)	G (98.5%, 978) S (0.4%, 4)	G (97.6%, 2602) S (0.1%, 4)
		1G7				
2	G132E	1H9	G (97.6%, 765) E (1.7%, 13)	G (99.2%, 883)	G (99.1%, 984)	G (98.7%, 2632) E (0.5%, 13)
3	T134I	5H8	T (89.2%, 699) I (0.6%, 8)	T (100%, 890)	T (99.4%, 987) I (0.1%, 1)	T (96.6%, 987) I (0.3%, 1)
4	S136N	2E7	S (44.5%, 349) N (49.9%, 391)	S (74.4%, 662) N (25.4%, 226)	S (94.8%, 941) N (2.3%, 23)	S (73.2%, 1952) N (24.0%, 640)
		2E9				
5	G151E	1D9	G (89.5%, 702) E (3.6%, 28)	G (97.5%, 868) E (0.6%, 5)	G (94.7%, 940) E (2.1%, 21)	G (94.1%, 2510) E (2.0%, 54)
		2D4				
		2D7				
		2G11				
		3C12				
		3E3				
		5D11				
		6F8				
6	G151E +R148M	1A8	R (56.5%, 423) M (2.8%, 22)	R (83.4%, 742) M (0.6%, 5)	R (81.1%, 805)	R (73.9%, 1970) M (1.0%, 27)
		1B4				
		2F10				
		3F3				
		5E9				
7	G151E +E113K	1D11	E (94.3%, 739)	E (99.1%, 882) K (0.8%, 7)	E (93.5%, 928) K (0.2%, 2)	E (95.6%, 2549) K (0.3%, 9)
8	N167D	2H9	N (99.5%, 780) D (0.3%, 2)	N (99.4%, 885) D (0.6%, 5)	N (99.3%, 986) D (0.3%, 3)	N (99.4%, 2651) D (0.4%, 10)
		3A9				
		3B10				
		3D11				
9	A168D	3C10	A (96.8%, 759) D (0.1%, 1)	A (99.8%, 888)	A (92.2%, 916) D (0.7%, 7)	A (96.1%, 2563) D (0.3%, 8)
10	M173K	1F6	M (96.6%, 757)	M (98%, 872)	M (95%, 943)	M (96.4%, 2572)
11	G214E	1B10	G (93.4%, 732) E (6.1%, 48)	G (97.1%, 864) E (0.7%, 6)	G (81.3%, 807) E (2.5%, 25)	G (90.1%, 2403) E (3.0%, 25)
		1C11				
		3C4				
12	G214E +R65K	2E10	R (86.6%, 679) K (11.1%, 87)	R (77.8%, 692) K (20.6%, 183)	R (88.9%, 883) K (7.7%, 76)	R (84.5%, 2254) K (13.0%, 346)

Note: H7 HA sequences were retrieved from GISAID (last accessed 30 March 2026). Percentages are normalized to the total number of sequences within each time period.

To determine whether the observed frequency shifts reflected directional evolution, temporal analysis focused on epitope sites with >5% variation across surveillance periods (S136, G214, and R65). The prevalence of S136 rose sharply from 44.5% (pre-2012) to 94.8% (2017–2025), a directional sweep confirmed by the Cochran–Armitage test (*P* < 0.0001). This fixation suggests that the ancestral residue may have carried a fitness cost under evolving host immune pressures, implying that mAbs targeting this site (e.g. 2E7 and 2E9) could exhibit renewed efficacy against contemporary H7 strains. Conversely, G214 and R65 displayed non-monotonic fluctuations with significant temporal heterogeneity (Pearson’s χ² test (both *P* < 0.0001), indicating cyclical immune selection or compensatory co-evolution.

### Antigenic and phylogenetic mutation profile

**Figure 3.**
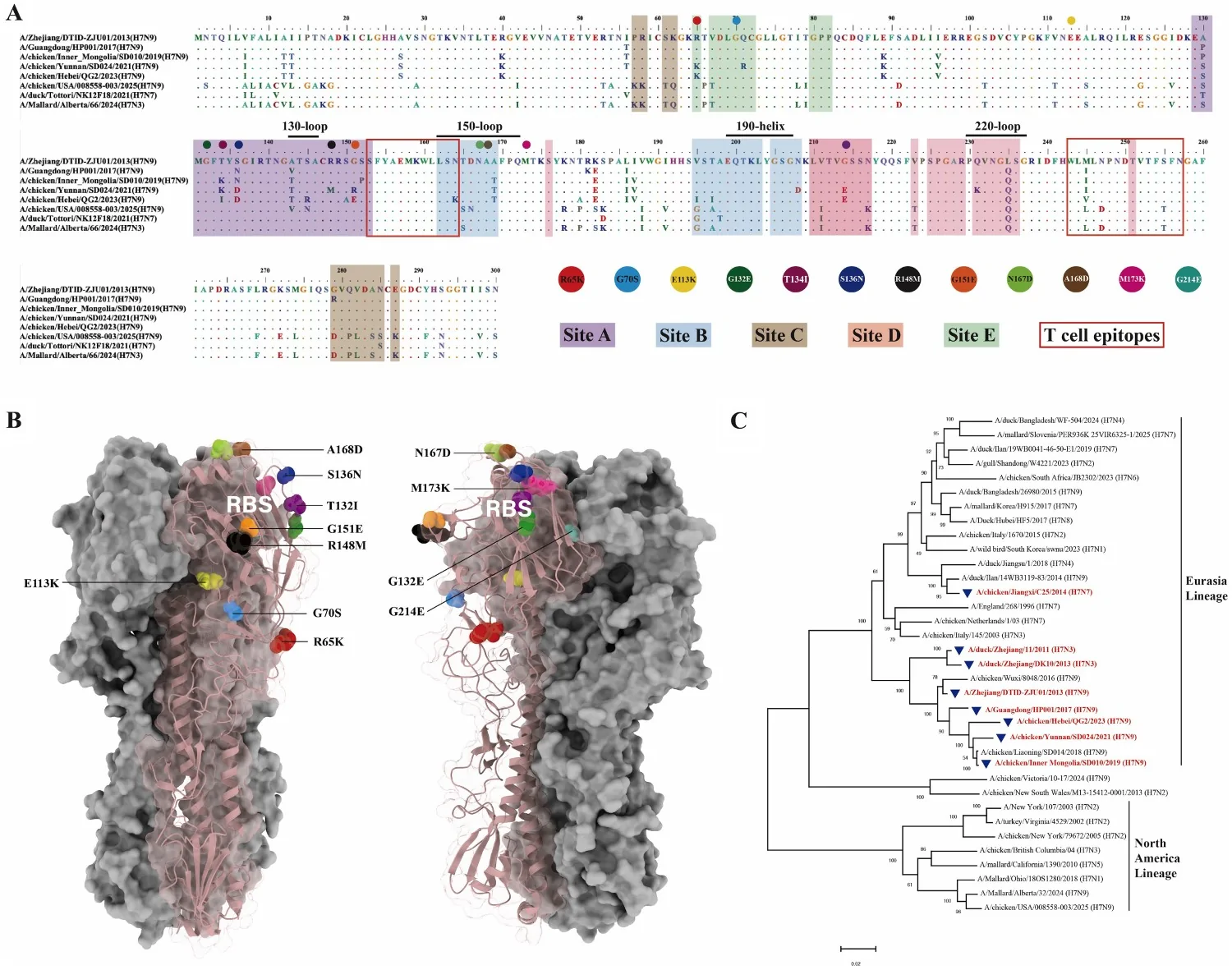
Structural mapping and phylogenetic analysis of H7 HA. (A) Amino acid sequence alignment of the HA1 domain across representative H7 strains, with classical antigenic sites (A–E) highlighted using distinct background colours (Site A, purple; Site B, blue; Site C, brown; Site D, pink; Site E, green); key structural elements of the receptor-binding domain, including the 130-loop, 150-loop, 220-loop, and 190-helix, are indicated above the alignment, along with previously characterized T-cell epitopes (red boxes), and specific amino acid substitutions are denoted by colour-coded circles. (B) Three-dimensional structural mapping of the identified mutations, in which residues shown in (A) were projected onto the H7 HA trimer (PDB: 4LN6) using PyMOL (v2.5.0), with mutations displayed as coloured spheres on a highlighted HA monomer (pink ribbon), illustrating their spatial distribution relative to the RBS on the HA head. (C) Phylogenetic relationships of H7 HA genes, reconstructed using the neighbor-joining method in MEGA, revealing distinct Eurasian and North American lineages; representative H7 viruses analyzed in this study are highlighted in red and marked with blue triangles. Bootstrap support values from 1,000 replicates are shown at the internal nodes. The scale bar represents 0.02 nucleotide substitutions per site.

Following the identification of these twelve escape sites, the key residues were mapped onto the HA sequences within the framework of five previously defined antigenic regions (A–E) [45], enabling a detailed characterization of the antigenic landscape (Figure 3A). Analysis of the HA head domain identified twelve substitutions strategically positioned to facilitate mAb evasion. These mutations were predominantly concentrated within immunodominant regions, particularly Site A (G132E, T134I, S136N, R148M, and G151E), which represents the principal hotspot for antigenic drift in H7 AIV and a major immunodominant region [45]. Specifically, S136N was centrally embedded within this classical B-cell antigenic site, while simultaneously mapping to a previously characterized T-cell epitope region [45]. Site B contained two critical substitutions (N167D and A168D) located within the 150-loop, a key structural component of the receptor-binding site (RBS). This distribution is consistent with the immunodominance of Site A- and Site B-targeting antibodies observed in human post-infection sera [46]. Additional substitutions were identified in Site E (R65 K and G70S) and Site D (G214E). Interestingly, mutations in different antigenic sites exhibited distinct evolutionary constraints. While variations in Site B are often associated with fitness costs, mutations in Site E, such as G70S, appear to be pathogenically neutral in avian models, suggesting differential selective pressures across antigenic regions [45]. Structural mapping onto the HA trimer (Figure 3B; PDB: 4LN6) revealed that most substitutions cluster around the periphery of the RBS, a topological arrangement that likely interferes with the binding of potent neutralizing antibodies. An exception was E113 K, which is located near the trimer interface. Phylogenetic analysis further demonstrated clear temporal and geographic clustering patterns among H7N9 viruses (Figure 3C).

### Characterization of chimeric BsAb-H7

**Figure 4.**
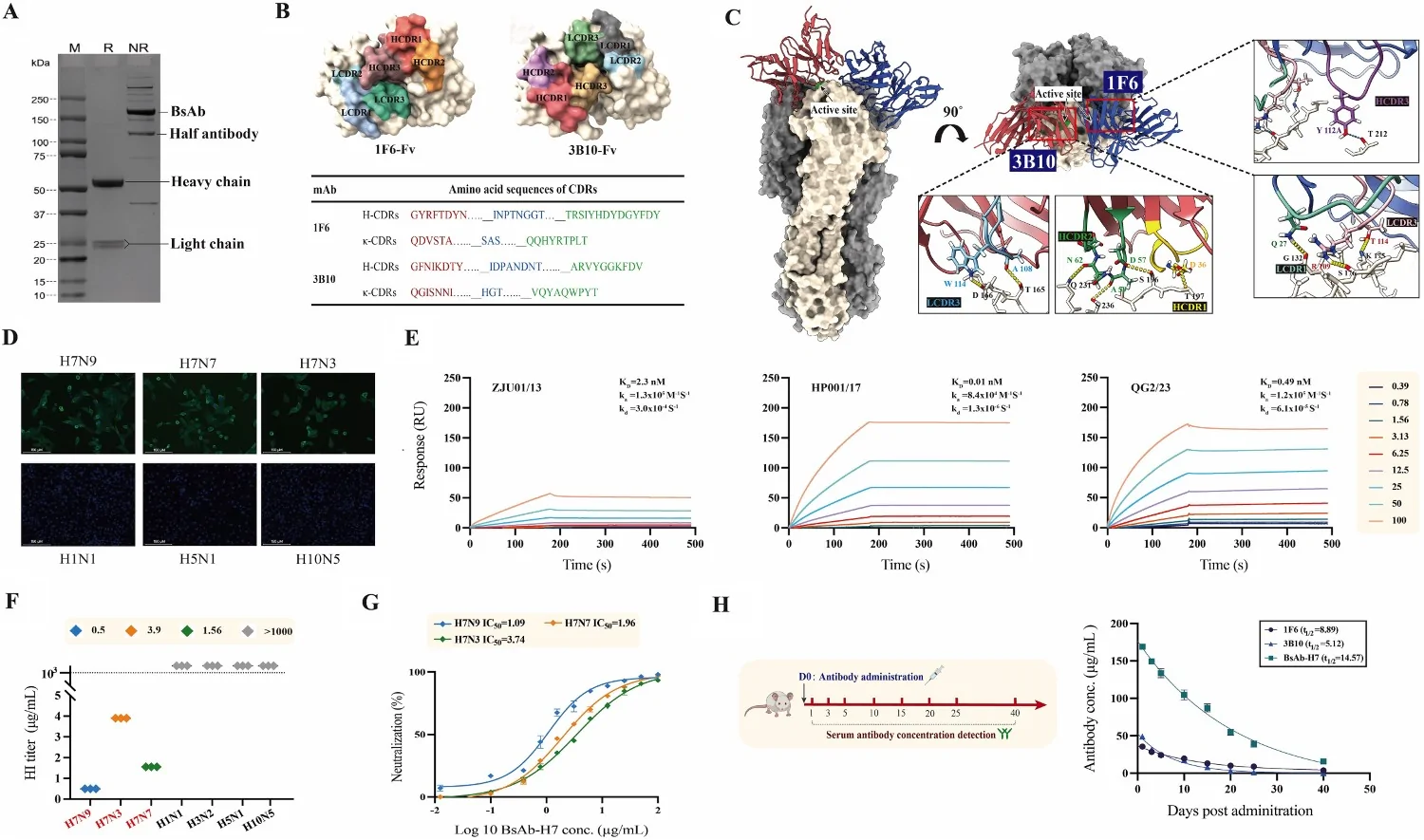
Comprehensive characterization and functional profiling of the BsAb-H7. (A) SDS-PAGE analysis assessing the purity and molecular assembly of BsAb-H7 under reducing (R) and non-reducing (NR) conditions. (B) Surface representations of the 1F6 and 3B10 Fv regions illustrating the spatial distribution of complementarity-determining regions (CDRs); in 1F6, HCDR1-3 are shown in coral, orange, and dusty pink, respectively, and LCDR1-3 in dusty blue, ice blue, and light sea green, whereas in 3B10, HCDR1-3 are coloured Indian red, purple, and brown, and LCDR1-3 are depicted in grey, powder blue, and greyish green; the corresponding amino acid sequences of each CDR are listed below the structures. (C) Molecular docking simulations illustrating bispecific engagement of BsAb-H7 with H7 HA, showing 1F6 (blue) and 3B10 (red) Fv domains bound to the surface-rendered HA trimer (PDB: 4LN6); magnified views reveal detailed intermolecular interactions, including hydrogen bonds (yellow dashed lines) between specific CDR loops and key HA residues (stick representation), with arrows indicating escape mutations identified during mAb selection, including M173 K (associated with 1F6) and N167D (associated with 3B10), localized within the predicted interfaces. (D) Immunofluorescence assay demonstrating binding of BsAb-H7 to MDCK cells infected with diverse H7 subtypes and non-H7 viruses, with green fluorescence (Alexa Fluor 488) indicating viral protein expression and blue fluorescence (DAPI) marking nuclei; scale bar = 150 μm. (E) SPR sensorgrams showing binding kinetics of BsAb-H7 to recombinant H7N9 HA proteins, with curves from a complete subset of concentrations (0.39-100 nM). Kinetic parameters are indicated above each sensorgram. (F) Hemagglutination inhibition activity of BsAb-H7 against H7N9 (blue), H7N7 (green), and H7N3 (yellow) strains, with no detectable activity against non-H7 subtypes (grey); titres (μg/mL) represent mean values from three independent experiments. (G) *In vitro* microneutralization curves demonstrating dose-dependent neutralizing activity against representative H7 strains, with IC_50_ values calculated from cell-based ELISA; data are presented as mean ± SD from at least two independent experiments. (H) *In vivo* pharmacokinetic profile of BsAb-H7, showing serum antibody decay over a 40-day period following a single 10 mg/kg administration in mice (n = 5 per group), with serum collected on days 1, 3, 5, 10, 15, 20, 25, and 40 and antibody concentrations determined by ELISA; the resulting clearance curves demonstrate an extended half-life of BsAb-H7 compared with its parental mAbs.

Based on the characterization and conservation analyses, and excluding epitope pairs prone to steric hindrance or conformational constraints [47], mAbs 1F6 and 3B10 were selected as optimal candidates. These antibodies were engineered into a bispecific format, designated BsAb-H7, comprising two independent antigen-binding arms fused to a common human Fc region to enhance stability and broaden antiviral breadth. Under reducing conditions, SDS-PAGE revealed distinct bands corresponding to the expected molecular weights of the heavy and light chains, with no evidence of degradation. Under non-reducing conditions, BsAb-H7 migrated as a single intact IgG species, indicating a stable disulfide-linked structure (Figure 4A). The amino acid sequences of the complementarity-determining regions (CDRs) within the heavy (HCDR1-3) and light (LCDR1-3) chains of each Fv arm are shown in Figure 4B, defining the paratope architecture responsible for dual specificity.

Molecular docking simulations were performed to predict the structural basis of 1F6 and 3B10 binding to the HA head (Figure 4C). The predicted binding mode of 1F6 indicates a network of polar interactions, with residues Q27 (LCDR1), R109, and T114 (LCDR3) interacting with HA residues G132, S176, and K175, respectively. In addition, Y112 in HCDR3 is predicted to form a polar interaction with HA residue T212. In contrast, 3B10 engages the HA head through a distinct set of interactions: light chain residues W114 and A108 (LCDR3) interact with D166 and T165, while heavy chain residues N62, A59, D57 (HCDR2), and D36 (HCDR1) interact with Q231, S236, S196, and T197, respectively. The spatial clustering of these sequence-dispersed HA residues on the globular head domain indicates that both 1F6 and 3B10 recognize discontinuous, conformational epitopes. These interfaces are further stabilized by extensive van der Waals interactions. Notably, escape mutations identified under mAb pressure, M173 K (for 1F6) and N167D (for 3B10), map within these predicted binding interfaces, supporting the structural epitope assignments and highlighting the functional relevance of these residues in antibody recognition.

BsAb-H7 demonstrated broad binding activity across a panel of homologous and heterologous H7 viruses (Figure 4D), while showing no detectable binding to non-H7 subtypes (H1N1, H3N2, H5N1, and H10N5). SPR analysis using recombinant HA proteins from temporally distinct H7N9 clades revealed high-affinity binding across all variants tested, with K_D_ values of 2.3 nM (ZJU01/13), 0.01 nM (HP001/17), and 0.49 nM (QG2/23) (Figure 4E), alongside corresponding k_a_ and k_d_ values as detailed in Figure 4E, indicating sustained recognition of evolving H7N9 strains. Consistently, BsAb-H7 exhibited potent HI activity exclusively against H7 viruses, with minimum inhibitory concentrations of 0.5 μg/mL for H7N9, 1.56 μg/mL for H7N7, and 3.9 μg/mL for H7N3, while no activity (>1000 μg/mL) was observed against other influenza A subtypes (Figure 4F). *In vitro* neutralization assays further confirmed dose-dependent antiviral activity, with IC_50_ values of 1.09, 1.96, and 3.74 μg/mL for H7N9, H7N7, and H7N3, respectively (Figure 4G), demonstrating broad neutralization within the H7 lineage.

In addition to its *in vitro* potency, the *in vivo* pharmacokinetic profile of BsAb-H7 was evaluated following a single administration in mice (Figure 4H). Longitudinal measurement of serum antibody levels over 40 days revealed a substantially prolonged half-life for BsAb-H7 compared with its parental antibodies. Specifically, BsAb-H7 exhibited a half-life (t_1/2_) of 14.57 days, exceeding that of 1F6 (8.89 days) and 3B10 (5.12 days). Concurrently, the bispecific antibody displayed significantly elevated systemic exposure (AUC) and reduced clearance (CL) compared with both 1F6 and 3B10 (*P* < 0.001; Table S2), indicating enhanced *in vivo* stability and potential for sustained therapeutic efficacy.

### Protective efficacy against H7N7 challenge

The prophylactic and therapeutic efficacy of BsAb-H7 was evaluated in a lethal H7N7 challenge model (5 × MLD_50_) and compared with its parental mAbs. Key clinical endpoints, including body weight and survival, were monitored daily. As extra-pulmonary viral titres (heart, spleen, kidneys, liver) were undetectable in all groups, lung viral loads were measured at 3 and 6 dpi to assess viral clearance across treatment groups.

**Figure 5.**
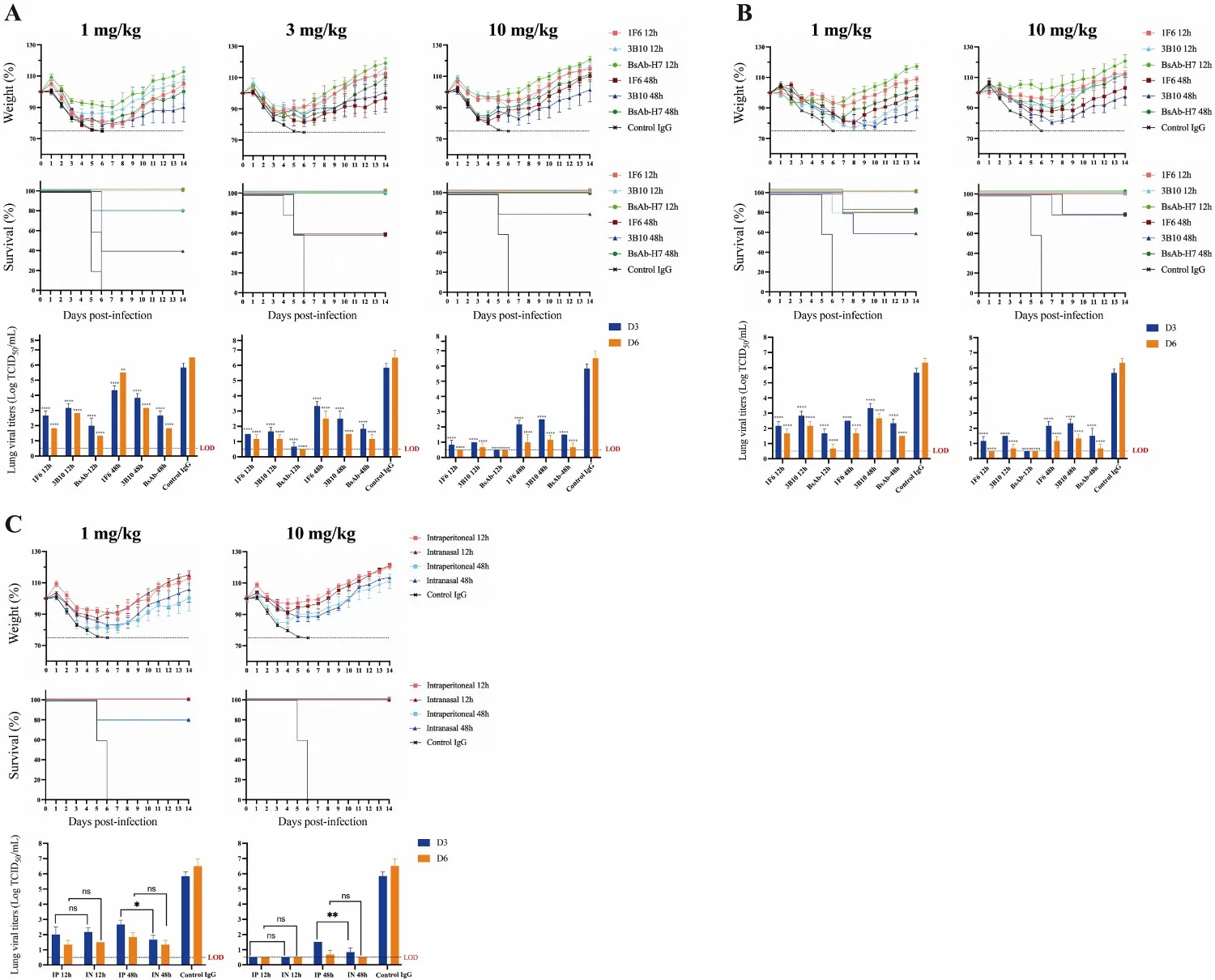
Protective efficacy and delivery routes of antibodies against lethal H7N7 challenge. Mice (n = 11 per group) were intranasally challenged with 5 × MLD_50_ of the H7N7 strain. (A) Therapeutic efficacy. Mice were treated with 1, 3, or 10 mg/kg of parental mAbs (1F6, 3B10), BsAb-H7, or isotype IgG control at 12 or 48 hpi. (B) Prophylactic efficacy. Mice were pre-treated IP with 1 or 10 mg/kg of the indicated antibodies at 12 or 48 h prior to infection. (C) Comparative therapeutic efficacy of IN versus IP administration. Mice were treated with BsAb-H7 via IN or IP route at 12 or 48 hpi, at doses of 1 or 10 mg/kg. For all panels, longitudinal body weight changes (top row) and Kaplan-Meier survival curves (middle row) were monitored over 14 days (n = 5 per group). Body weights are expressed as a percentage of the initial weight (mean ± SD). Lung viral titres (bottom row) were determined by TCID_50_ assay at 3 and 6 dpi from euthanized mice (n = 3 per group). Data are presented as mean ± SD, with horizontal dashed lines indicating the limit of detection (LOD, red). Statistical significance for viral titres was determined by two-way ANOVA with multiple comparisons. In (A) and (B), significance is indicated compared with time-matched isotype controls (**P < 0.01, ****P < 0.0001). In (C), statistical comparisons were analyzed between IN and IP administration at matched time points (*P < 0.05, **P < 0.01; ns, no significant difference).

In therapeutic settings, BsAb-H7 provided complete protection against morbidity and mortality across all tested doses (1, 3, and 10 mg/kg) and treatment time points (12 and 48 hpi), whereas all control mice succumbed to infection by 6 dpi (Figure 5A). The efficacy of both parental mAbs and BsAb-H7 exhibited dose- and time-dependent effects. At 12 hpi, 1F6 achieved 100% survival at 1 mg/kg, while 3B10 required a higher dose of 3 mg/kg to reach comparable protection. When treatment was delayed to 48 hpi, 1F6 required 10 mg/kg to maintain full protection, whereas 3B10 achieved only 80% survival. In contrast, BsAb-H7-treated mice displayed minimal weight loss and significantly improved clinical outcomes compared with parental antibody groups. These findings were consistent with virological data; notably, BsAb-H7 reduced lung viral titres to below the limit of detection (LOD) as early as 3 dpi at doses ≥3 mg/kg in the 12-hpi cohort, indicating rapid and effective viral clearance.

Prophylactic efficacy was similarly assessed using the same H7N7 challenge model (Figure 5B). At 12 h prior to infection, both BsAb-H7 and 1F6 conferred complete protection across all tested doses, whereas 3B10 achieved full protection only at 10 mg/kg. When administered 48 h prior to challenge, BsAb-H7 maintained 100% survival at 10 mg/kg, outperforming both 1F6 and 3B10, which each achieved 80% survival. Consistent with clinical outcomes, lung viral loads were significantly reduced in BsAb-H7-treated mice. Collectively, these results demonstrate that BsAb-H7 provides superior therapeutic and prophylactic efficacy against lethal H7N7 infection.

### Intranasal BsAb-H7 elicits protective responses in the H7N7 mouse model

Given that influenza primarily targets the respiratory tract, we compared mucosal IN delivery with conventional systemic IP administration to evaluate their relative protective efficacy. Following lethal H7N7 challenge, mice were treated with BsAb-H7 via IN or IP routes at 12 or 48 hpi. Both administration routes conferred robust and comparable survival benefits. Early intervention (12 hpi) at 10 mg/kg resulted in complete (100%) protection, whereas delayed treatment (48 hpi) at a lower dose (1 mg/kg) achieved 80% survival, regardless of the delivery route (Figure 5C).

Despite similar survival outcomes, longitudinal body weight analysis revealed a clear therapeutic advantage of localized IN delivery under delayed treatment conditions. While IN and IP administration at 12 hpi similarly mitigated disease severity, IN treatment at 48 hpi more effectively attenuated morbidity than IP administration. Specifically, mice receiving IN treatment exhibited a less pronounced weight loss and a faster recovery trajectory across both dose groups compared with IP-treated counterparts. This enhanced clinical benefit was further supported by virological data: TCID_50_ analysis demonstrated that delayed IN administration (1 and 10 mg/kg) significantly reduced lung viral loads (*P* < 0.05 and *P* < 0.01, respectively, compared with the IP-treated groups), confirming its superior local antiviral efficacy.

### Histological analysis

**Figure 6.**
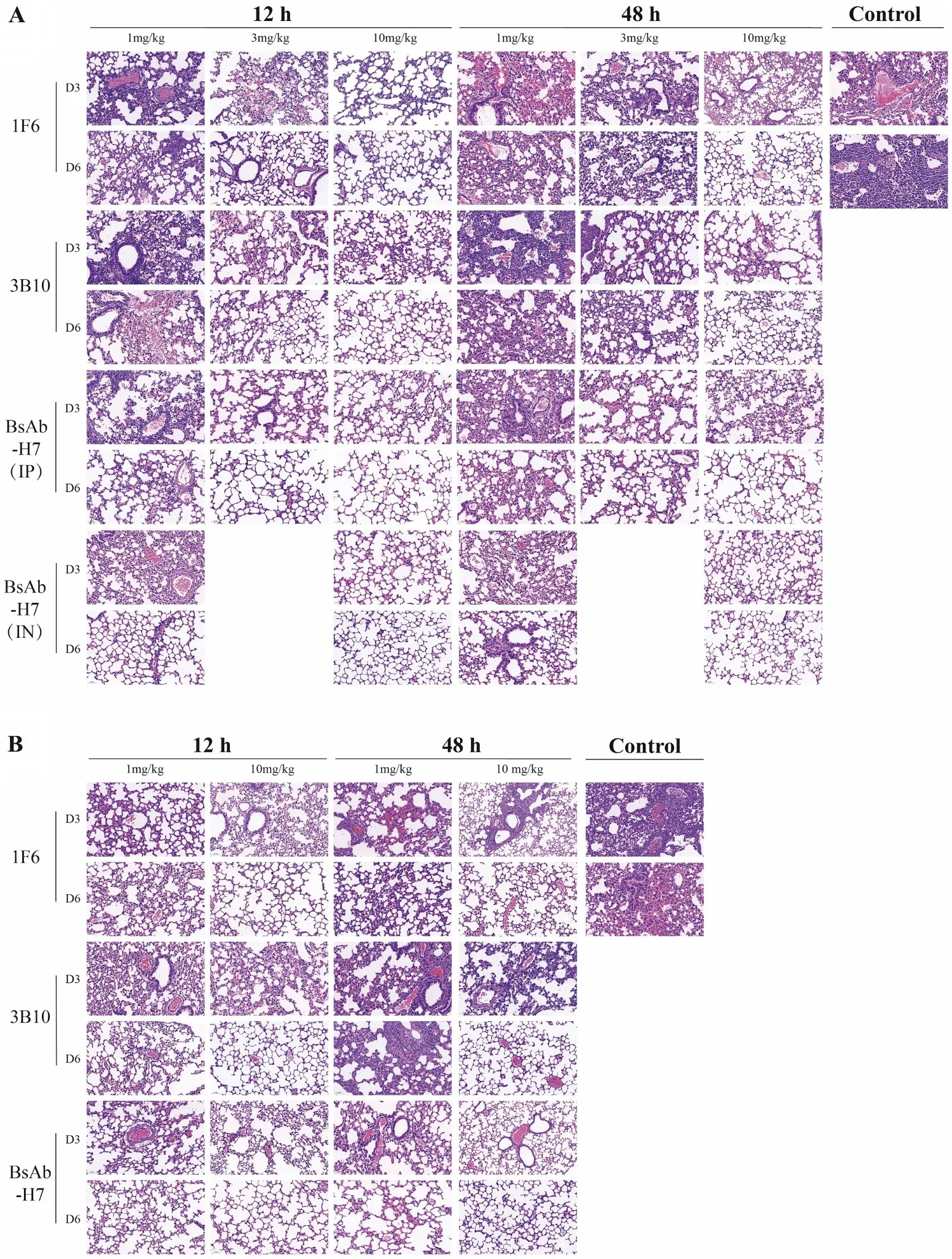
Histopathological evaluation of lung tissues following therapeutic and prophylactic antibody administration. (A) Representative hematoxylin and eosin (H&E)-stained lung sections illustrating therapeutic effects of parental mAbs (1F6 and 3B10) and BsAb-H7 following H7N7 infection; mice were treated via IP or IN routes at 12 or 48 hpi at the indicated doses, and lung tissues were collected at 3 and 6 dpi (D3 and D6). (B) Representative H&E-stained lung sections demonstrating prophylactic protection in mice pre-treated with 1 or 10 mg/kg of the indicated antibodies at 12 or 48 h prior to infection.

To further elucidate the mechanisms underlying the robust clinical protection and viral clearance conferred by BsAb-H7, we performed a comprehensive histopathological analysis of lung tissues using hematoxylin and eosin staining. In untreated control mice, H7N7 infection induced severe and progressive pulmonary damage. By 3 and 6 dpi, lung tissues exhibited extensive inflammatory cell infiltration, marked alveolar septal thickening, perivascular cuffing, and hemorrhage. Antibody treatment markedly alleviated these pathological changes in a dose- and time-dependent manner (Figure 6A). Consistent with survival and viral load data, mice treated with parental mAbs (1F6 and 3B10) showed only partial resolution of inflammation, with residual interstitial infiltrates persisting at lower doses (1 mg/kg) or with delayed administration (48 hpi). In contrast, systemic IP administration of BsAb-H7 largely preserved alveolar architecture and minimized inflammatory exudation across all conditions tested, supporting its superior therapeutic efficacy.

We next compared tissue-level outcomes between mucosal and systemic delivery routes. Histological examination demonstrated that early intervention (12 hpi) via either route effectively prevented severe lung injury. However, under delayed treatment conditions (48 hpi), IN administration conferred a clear advantage over IP delivery. Lungs from IN-treated mice exhibited more rapid resolution of peribronchial and interstitial inflammation, consistent with the improved clinical recovery observed in these animals.

Parallel histological analyses were conducted to evaluate prophylactic efficacy (Figure 6B). Pre-treatment effectively protected lung tissue from infection-induced structural damage. Parental mAbs required higher doses to fully prevent diffuse alveolar injury, particularly when administered 48 h prior to infection. In contrast, BsAb-H7 provided robust tissue protection across all tested conditions, with lung sections consistently displaying well-preserved alveolar spaces even under stringent experimental settings. These findings provide strong histopathological evidence that BsAb-H7 not only suppresses viral replication but also mitigates virus-induced lung injury, highlighting its potential as both a prophylactic and therapeutic agent against H7N7 infection.

## Discussion

Despite the widespread use of bivalent poultry vaccines, the persistent cryptic circulation and ongoing antigenic drift of H7 avian influenza viruses continue to pose a substantial pandemic threat [48,49]. In this context, the identification of conserved, functionally constrained epitopes on HA remains a critical objective for the development of effective countermeasures.

In this study, by systematically mapping the antigenic architecture of the H7 HA globular head, we identified Site A as the dominant immunogenic region, accounting for 42% (5 of 12) of the identified escape substitutions. Notably, Site A contains highly conserved motifs shared across pathogenic H7 lineages, making it a key target for subtype-specific broadly neutralizing antibodies [50].

Among the 12 mapped epitopes, seven residues exhibited conservation rates exceeding 95%, with four (G70S, G132E, N167D, and M173 K) remaining nearly invariant over extended evolutionary timescales despite ongoing immune pressure. The rare occurrence of escape mutations at these sites suggests that alterations may incur substantial fitness costs, potentially compromising HA structural stability or receptor-binding function [51]. Exploiting these conserved vulnerabilities confers broad-spectrum protection while fully preserving the robust hemagglutination inhibition (HI) activity that is typically compromised in stem-directed interventions, thereby establishing these sites as highly promising targets for next-generation universal biologics.

To overcome the inherent vulnerability of monospecific targeting to viral escape [35], we engineered BsAb-H7 to simultaneously engage two spatially distinct, structurally constrained epitopes. Notably, this design diverges from conventional HA-targeting BsAbs that empirically pair head- and stem-specific clones to balance potency and breadth. Instead, our dual-head architecture was rationally guided by evolutionary profiling of >2,500 H7 isolates, which identified two stringently conserved epitopes (>95% global conservation). This dual-head strategy offers two distinct advantages over head-and-stem configurations. First, by directly blocking sialic acid receptor engagement, head-directed paratopes achieve superior, largely Fc-independent neutralization potency, circumventing the reliance on FcγR-mediated effector functions required by stem-directed antibodies *in vivo* [52]. Moreover, because both targeted loci reside within critical regions of the receptor-binding site, viral escape necessitates concurrent mutations at both sites, imposing a prohibitive fitness cost [14]. In addition to limiting escape, the physical linkage of the 1F6 and 3B10 variable domains confers synergistic effects through enhanced avidity. Consistent with this design, BsAb-H7 exhibited robust *in vitro* neutralization across diverse H7 subtypes (H7N9, H7N7, and H7N3), yielding IC_50_ values of 1.09-3.74 μg/mL and potent HI activity at concentrations of 0.5-3.9 μg/mL. While the neutralization potency against H7N9 is comparable to that of established H7 head-specific antibodies, with an IC_50_ of approximately 1 μg/mL [5], BsAb-H7 affords substantially broader cross-subtype coverage. SPR analysis further demonstrated exceptionally high binding affinity across temporally distinct H7N9 clades, with K_D_ values ranging from 0.01 to 2.3 nM, indicating sustained recognition despite ongoing viral evolution. Furthermore, the bispecific format also conferred an enhanced *in vivo* pharmacokinetic profile, characterized by significantly increased systemic exposure, reduced clearance, and a prolonged serum half-life (t_1/2_ = 14.57 days), substantially exceeding that of the parental mAbs, 1F6 (8.89 days) and 3B10 (5.12 days). These findings highlight how rational, structure-guided bispecific engineering translates epitope conservation into enhanced molecular potency and improved *in vivo* stability.

BsAb-H7 also demonstrated robust *in vivo* efficacy in both prophylactic and therapeutic settings. Importantly, IN delivery accelerated viral clearance and resolved pulmonary pathology more rapidly than systemic administration under delayed treatment conditions. This localized advantage likely reflects the immediate availability of neutralizing antibodies at the primary site of viral replication, thereby limiting early viral expansion and systemic dissemination [53,54]. Notably, this benefit is not unique to BsAb-H7: intranasal delivery has similarly enhanced the efficacy of bispecific antibodies against SARS-CoV-2 [55] and RSV [56], indicating a generalizable synergy between bispecific formats and mucosal delivery. Our study extends this principle to post-exposure influenza intervention, highlighting a critical clinical advantage. While IgG-based antibodies, including BsAb-H7, are inherently less effective than secretory IgA at preventing initial viral entry [57], this limitation aligns with its therapeutic positioning. The dual-epitope potency of BsAb-H7, combined with its compatibility with non-invasive mucosal delivery, provides a translationally attractive framework for pandemic response.

Despite the promising therapeutic potential of BsAb-H7, several limitations should be acknowledged. First, although dual targeting of highly conserved epitopes is designed to elevate the genetic barrier to viral escape, we did not perform serial passage under antibody pressure. Future *in vitro* forced-escape assays, integrated with genomic surveillance of these loci in circulating strains, are required to definitively establish the resistance profile of BsAb-H7. Additionally, while our computational docking models are consistent with empirical escape mutation profiles, the lack of high-resolution structural data (e.g. cryo-EM or X-ray crystallography) or site-directed mutagenesis precludes atomic-level characterization of the BsAb-HA interface. Subsequent studies employing reverse genetics and structural biology will be essential to precisely map binding energetics and guide further rational optimization.

Importantly, our *in vivo* lethal challenge experiments utilized a heterologous H7N7 surrogate model rather than the homologous H7N9 strain used for antibody generation. While this divergent challenge compellingly underscores the cross-lineage protective efficacy of BsAb-H7, it leaves the direct *in vivo* potency against contemporary H7N9 isolates unexplored. To reconcile this, our evolutionary and *in vitro* analyses verified that the targeted hemagglutinin head epitopes are highly conserved and functionally vulnerable across divergent H7 lineages. This cross-protection is further supported by our prior demonstration of *in vivo* efficacy against H7N9 using phylogenetically related antibodies [24]. Nevertheless, *in vivo* evaluation of BsAb-H7 against contemporary H7N9 isolates remains necessary for further preclinical validation.

Regarding the clinical translation of BsAb-H7, the potential immunogenicity associated with its chimeric format should be considered. However, unlike chronic diseases requiring long-term administration, influenza is an acute and self-limiting infection, which substantially reduces the risk associated with transient antibody exposure. Pharmacokinetic analyses showed that BsAb-H7 exhibits a serum half-life of approximately 14 days, indicating that a single-dose regimen may provide sufficient therapeutic coverage. Such a strategy is expected to minimize anti-drug antibody formation while avoiding the cumulative toxicities associated with repeated administration of murine-derived sequences. The clinical feasibility of this approach has been demonstrated in other acute viral settings, including the chimeric antibody cocktail ZMapp developed for Ebola virus disease [58].

Nevertheless, the current chimeric construct serves as an intermediate stage in therapeutic development [59]. Definitive clinical translation will necessitate humanization of BsAb-H7 to mitigate the residual immunogenicity. A feasible strategy involves CDR grafting onto human germline frameworks followed by affinity maturation to restore potential losses in binding or neutralizing activity, as exemplified by clinically approved bispecific antibodies such as Emicizumab [60]. In parallel, although intranasal administration of the IgG-based BsAb-H7 demonstrated substantial efficacy, the underlying mucosal pharmacokinetics and local immune responses within the respiratory tract require further investigation [57]. Future preclinical studies will systematically address these remaining translational hurdles, including the regulatory pathways for mucosal delivery and comparative evaluations against approved antivirals.

## Conclusions

In summary, this study provides a comprehensive characterization of 30 murine mAbs targeting H7 HA, delineating the antigenic architecture of the HA head domain. By integrating the complementary specificities of two lead candidates, we developed a bispecific antibody, BsAb-H7, that synergistically enhances the neutralization breadth and potency of the parental mAbs. These findings demonstrate that bispecific targeting of conserved epitopes represents a rational and effective strategy to counteract antigenic drift and develop broadly protective interventions against emerging influenza viruses.

## Supplementary Material

Supplementary Tables.docx

## Data Availability

The datasets used and/or analyzed during the current study are available from the corresponding author upon reasonable request.
