# Supplementary material for: A bispecific antibody targeting conserved hemagglutinin epitopes confers broad protection against H7 avian influenza and accelerates viral clearance via localized respiratory delivery
Source: Emerg Microbes Infect. 2026 Aug 19;15(1):2713326. doi: 10.1080/22221751.2026.2713326 (PMC13491902; doi:10.1080/22221751.2026.2713326)
Supplement: Supplementary file 1 [file TEMI_A_2713326_SM6698.docx]

**Table S1. Viruses included in the study**

| **Class** | **Strain** | **Subtype** |
| --- | --- | --- |
| H7 viruses | A/Zhejiang/DTID-ZJU01/2013 | H7N9  H7N9 |
|  | A/Guangdong/HP001/2017 |  |
|  | A/chicken/Jiangxi/C25/2014 | H7N7 |
|  | A/duck/Zhejiang/DK10/2013 | H7N3 |
| Other influenza viruses | A/Michigan/45/2015 | H1N1 |
|  | A/duck/Zhejiang/6D10/2013 | H2N8 |
|  | A/Hong Kong/2671/2019 | H3N2 |
|  | A/Texas/37/2024 | H5N1 |
|  | A/chicken/Zhejiang/1664/2017 | H6N1 |
|  | A/duck/Zhejiang/D4/2018 | H9N2 |
|  | A/Zhejiang/CNIC-ZJU01/2023 | H10N5 |
|  | B/Phuket/3073/2013 | Influenza B virus |

**Table S2. Pharmacokinetic parameters of BsAb-H7 and parental antibodies in mice following a single intraperitoneal dose of 10 mg/kg (n=5)**

| **Parameters** | **BsAb-H7** | **1F6** | **3B10** | **Unit** |
| --- | --- | --- | --- | --- |
| t_1/2_ | 14.57**/*** | 8.89 | 5.12 | days |
| AUC_0_–t | 2675.05*** | 508.29 | 341.07 | μg·day/mL |
| AUC_0_–∞ | 2928.16*** | 579.19 | 352.04 | μg·day/mL |
| CL | 3.42*** | 28.43 | 17.28 | mL/day/kg |
| Vd | 54.66*** | 324.62 | 152.84 | mL/kg |

Note: Statistical analyses were performed using one-way ANOVA followed by Tukey's *post-hoc* test. Significance levels are indicated as follows: ****P* < 0.001 for BsAb-H7 versus both control groups, and ***P* < 0.01 for BsAb-H7 versus the 1F6 group in half-life (t_1/2_) assessment. Pharmacokinetic parameters are defined as: AUC_0_–t, area under the concentration-time curve from zero to the last measurable time point; AUC_0_-∞, area under the curve extrapolated to infinity; CL, apparent clearance; Vd, apparent volume of distribution.
